# Mid-term treatment-related cognitive sequelae in glioma patients

**DOI:** 10.1007/s11060-022-04044-1

**Published:** 2022-07-07

**Authors:** Sabine Schlömer, Jörg Felsberg, Milena Pertz, Bettina Hentschel, Markus Löffler, Gabriele Schackert, Dietmar Krex, Tareq Juratli, Joerg Christian Tonn, Oliver Schnell, Hartmut Vatter, Matthias Simon, Manfred Westphal, Tobias Martens, Michael Sabel, Martin Bendszus, Nils Dörner, Klaus Fliessbach, Christian Hoppe, Guido Reifenberger, Michael Weller, Uwe Schlegel, for the German Glioma Network

**Affiliations:** 1 Department of Neurology, University Hospital Knappschaftskrankenhaus, Ruhr University Bochum, In der Schornau 23-25, D-44892 Bochum, Germany; 2 Institute of Neuropathology, Heinrich Heine University Medical Faculty and University Hospital Düsseldorf, Düsseldorf, Germany; 3 Institute for Medical Informatics, Statistics and Epidemiology, University of Leipzig, Leipzig, Germany; 4 Department of Neurosurgery, University Hospital, University Carl Gustav Carus of Dresden, Dresden, Germany; 5 Department of Neurosurgery, University Hospital, Ludwig Maximilians University of Munich, Munich, Germany; 6 Department of Neurosurgery, Medical Center - University of Freiburg, Freiburg, Germany; 7 Department of Neurosurgery, University Hospital Bonn, Bonn, Germany; 8 Department of Neurosurgery, Medical Center Bethel, University Hospital Bielefeld, Bielefeld, Germany; 9 Department of Neurosurgery, University Hospital Hamburg-Eppendorf, Hamburg, Germany; 10 Department of Neurosurgery, Medical Center Asklepios St. Georg, Hamburg, Germany; 11 Department of Neurosurgery, Heinrich Heine University Medical Faculty and University Hospital Düsseldorf, Düsseldorf, Germany; 12 Department of Neuroradiology, Medical Center of Neurology, University Hospital Heidelberg, Heidelberg, Germany; 13 Institute for Diagnostic and Interventional Radiology and Neuroradiology, University Hospital Essen, Essen, Germany; 14 Department of Neurodegenerative Diseases and Geriatric Psychiatry, University Hospital Bonn, Bonn, Germany; 15 Department of Epileptology, University Hospital Bonn, Bonn, Germany; 16 Department of Neurology, University Hospital and University of Zurich, Zurich, Switzerland; 17 Department of General Neurology, University Hospital Tübingen, Tübingen, Germany

**Keywords:** Glioma, Neuropsychological assessment, NeuroCog FX, Prospective, Treatment-related neurotoxicity

## Abstract

**Purpose:**

Cognitive functioning represents an essential determinant of quality of life. Since significant advances in neuro-oncological treatment have led to prolonged survival it is important to reliably identify possible treatment-related neurocognitive dysfunction in brain tumor patients. Therefore, the present study specifically evaluates the effects of standard treatment modalities on neurocognitive functions in glioma patients within two years after surgery.

**Methods:**

Eighty-six patients with World Health Organization (WHO) grade 1–4 gliomas were treated between 2004 and 2012 and prospectively followed within the German Glioma Network. They received serial neuropsychological assessment of attention, memory and executive functions using the computer-based test battery NeuroCog FX. As the primary outcome the extent of change in cognitive performance over time was compared between patients who received radiotherapy, chemotherapy or combined radio-chemotherapy and patients without any adjuvant therapy. Additionally, the effect of irradiation and chemotherapy was assessed in subgroup analyses. Furthermore, the potential impact of the extent of tumor resection and histopathological characteristics on cognitive functioning were referred to as secondary outcomes.

**Results:**

After a median of 16.8 (range 5.9–31.1) months between post-surgery baseline neuropsychological assessment and follow-up assessment, all treatment groups showed numerical and often even statistically significant improvement in all cognitive domains. The extent of change in cognitive functioning showed no difference between treatment groups. Concerning figural memory only, irradiated patients showed less improvement than non-irradiated patients (*p* = 0.029, *η*^*2*^ = 0.06). Resected patients, yet not patients with biopsy, showed improvement in all cognitive domains. Compared to patients with astrocytomas, patients with oligodendrogliomas revealed a greater potential to improve in attentional and executive functions. However, the heterogeneity of the patient group and the potentially selected cohort may confound results.

**Conclusion:**

Within a two-year post-surgery interval, radiotherapy, chemotherapy or their combination as standard treatment did not have a detrimental effect on cognitive functions in WHO grade 1–4 glioma patients. Cognitive performance in patients with adjuvant treatment was comparable to that of patients without.

**Supplementary Information:**

The online version contains supplementary material available at 10.1007/s11060-022-04044-1.

## Introduction

Since significant advances in early diagnosis and efficient treatment of gliomas [1, 2] have led to prolonged overall survival, possible late treatment effects become increasingly important. As cognitive functioning represents a determinant of quality of life (QoL) it is important to reliably identify possible treatment-related neurocognitive dysfunction.

Although radiotherapy (RT) is thought to significantly contribute to long-term neurocognitive deterioration [3–6], it is unclear to what extent posttherapeutic deficits are caused by RT itself or by confounding factors such as the tumor, disease progression, surgery, antiepileptic drugs (AED) or treatment variables (e.g. radiation dosage and technique). Neurocognitive impairment, especially in verbal delayed recall was present in a heterogeneous group of brain tumors 18 months after fractionated stereotactic RT including the hippocampus [7]. Within the pivotal Radiation Therapy Oncology Group (RTOG) 98-02-trial, the addition of procarbazine, lomustine and vincristine (PCV) chemotherapy to RT revealed a several years increase of progression-free and overall survival in World Health Organization (WHO) grade 2 gliomas without higher rates of cognitive decline in the combined treatment group as compared to RT alone within five-years follow-up [8]. However, cognitive function was evaluated using the Mini-Mental-Status-Examination (MMSE) only [9, 10].

In a retrospective multicenter study, cognitive decline in 195 “low-grade glioma” (LGG) patients (recruited between 1997 and 2000) at median of six years post-RT was primarily attributed to the tumor itself or to single radiation fraction doses > 2 Gy [11]. However, at very long-term follow-up, (progressive) cognitive decline was present even for single fraction doses ≤ 2 Gy at median 12 years after RT in 65 progression-free patients [12]. Since recruitment was retrospective and treatment of irradiated patients dated back to the 1980s the grading system applied to this series did not incorporate molecular genetics. In the largest multicenter prospective, randomized controlled trial (European Organization for Research and Treatment of Cancer, EORTC 22033-26033) [13] no detrimental effect of RT on health-related QoL or MMSE scores was documented during the first three years of follow-up when comparing “LGG” patients treated with temozolomide chemotherapy alone or RT alone. However, since no comprehensive cognitive testing was used, subtle changes were not evaluated [14]. Analysis of isocitrate dehydrogenase (IDH) 1/2 wildtype versus mutant status revealed profound impact on prognosis [13], but further subgroup analyses with respect to neurocognition have not been carried out [14]. Published clinical studies on neurocognitive functioning after RT generally evaluated small patient cohorts and/or were based on retrospective analyses [3–6], applied cognitive screenings [10, 15] or questionnaires [16], included high single fraction doses (> 2 Gy) [4] or whole brain radiotherapy (WBRT) [4, 5]. Methodically high quality analyses with comprehensive neurocognitive batteries rarely exceeded few months of follow-up [17–19].

The present analysis represents the “mid-term” prospective evaluation of treatment-related neurocognitive sequelae in glioma patients within two years after surgery as a primary outcome, in a well-documented patient cohort undergoing serial neuropsychological testing for several years.

## Patients and methods

### Patients

In this multicenter longitudinal study, adult patients from the German Glioma Network were prospectively included between 2004 and 2012. Tumor classification and WHO grading were carried out prior to the revised 4th edition of the WHO classification of tumors of the central nervous system (CNS) [20] such that patients were categorized according to the WHO grading system for CNS tumors in its versions of 2000 [21] and 2007 [22] with therapeutic implication, i.e. WHO grade 2 gliomas were not irradiated upfront. In order to achieve the best possible adaptation of analyses to the current tumor classification system, all available tumor samples were reinvestigated according to the 5th edition of the WHO classification of CNS tumors published in 2021 [1], considering their IDH wildtype versus mutant status and presence or absence of 1p/19q co-deletion. Information on molecular genetics was completed in 61 of 86 tumors (Table 1). Patients had been enrolled in university hospitals of Dresden, Munich, Bonn, Hamburg, Düsseldorf and Bochum, Germany. This study was performed in accordance with the ethical standards laid down in the 1964 Declaration of Helsinki and approved by the local ethics committees and the ethics committee of the leading institution in Tübingen, Germany (Registration No.: 353/2003V). All patients gave written informed consent. Patients were excluded if they suffered from aphasia, psychosis or dementia prior to glioma diagnosis or if they had MMSE [23] scores < 20.

**Table 1:** Clinical and sociodemographic characteristics of patients, separated for treatment groups

	Treatment						
	Radiotherapy (RT)	Combined Radio-Chemotherapy (RChT)	Chemotherapy (ChT)	Watch-and-wait			
	n = 10	n = 24	n = 21	n = 31	p-value (comparison RT, RChT, ChT, watch-and-wait)	p-value (comparison RT+, RT-)	p-value (comparison ChT+, ChT-)
**Median age in years (range) at surgery**	25.8 (18.3–50.1)	38.0 (28.1–55.0)	38.4 (20.2–52.4)	30.8 (19.5–54.6)	0.02*	0.289	0.003**
**Sex, n, female : male**	5:5 (50%:50%)	10:14 (42%:58%)	7:14 (33%:67%)	17:14 (55%:45%)	0.463	0.515	0.104
**Education in years**							
Mean (SD)	12.5 (1.7)	11.8 (1.9)	12.1 (1.9)	11.7 (2.0)	0.603	0.716	0.957
**Surgery, n**					0.019* ^c^	0.324 ^c^	0.527 ^c^
Gross total resection^a^	3	9	7	15			
Subtotal resection^a^	1	9	4	9			
Partial resection^a^	2	5	3	3			
Biopsy (open vs. stereotactic)	4 (1 vs. 3)	1 (stereotactic)	7 (2 vs. 5)	4 (1 vs. 3)			
**Tumor histology (re-classified according to the revised WHO classification 2021 [**1**]), n**							
Astrocytoma, IDH-mutant	3	13	6	11	0.154 ^d^	0.190 ^d^	0.117 ^d^
Oligodendroglioma, IDH-mutant and 1p/19q-codeleted	–	4	9	5			
Glioblastoma, IDH-wildtype	–	2	–	–			
Other	1 (ganglioglioma)	–	–	7 (3 pilocytic astrocytoma, 2 ependymoma, 1 subependymoma, 1 ganglioglioma)			
Not re-classified	6	5	6	8			
**WHO grade according to the revised WHO classification 2021** [1**], n**							
WHO grade 1	1	–	–	5			
WHO grade 2	–	7	6	17 (2 ependymoma)	0.002** ^e^	0.147 ^e^	0.034* ^e^
WHO grade 3	3	5	9	1			
WHO grade 4	–	7	–	–			
Not re-classified	6	5	6	8			
**IDH1/2 mutation status, n**							
Wildtype	–	2	–	–			
Mutant	3	17	15	16			
Not done^b^	1	–	–	7			
Not re-classified	6	5	6	8			
**1p/19q-codeletion, n**							
Yes	–	4	9	5			
No	3	15 (2 glioblastoma)	6	11			
Not done^b^	1	–	–	7			
Not re-classified	6	5	6	8			
**Lateralization of tumor, n**							
Left	8	11	11	13	0.039* ^f^	0.218 ^f^	0.313 ^f^
Right	–	13	10	16			
Bilateral	2	–	–	–			
Crossing midline	–	–	–	1			
Other	–	–	–	1			
**Localization of tumor, n**							
Frontal	2	7	8	14	0.429 ^g^	0.102 ^g^	0.373 ^g^
Temporal	3	9	7	6	0.487 ^h^	0.216 ^h^	0.125 ^h^
Parietal	2	–	2	1			
Fronto-temporal	–	5	1	1			
Temporo-parietal	–	2	–	1			
Parieto-occipital	–	–	1	1			
Multifocal	–	1	–	1			
Other	3	–	2	6			
**AED at T1, n**	7	14	12	13	0.377	0.153	0.268
**AED at T2, n**	5	9	12	12	0.498	0.409	0.395
**Time interval T1 – T2 in mo**							
Mean (SD)	17.1 (7.4)	19.4 (7.1)	17.1 (6.7)	15.6 (5.9)	0.264	0.111	0.113
Median (range)	18.1 (5.9–28.1)	21.1 (8.0–31.1)	17.8 (6.2–28.4)	15.2 (6.1–24.3)			
**Time interval surgery – T1 in mo**							
Mean (SD)	0.3 (0.2)	0.2 (0.1)	0.3 (0.2)	0.6 (1.0)	0.502	0.607	0.840
Median (range)	0.3 (0.03–0.9)	0.2 (0.03–0.6)	0.2 (0.1–0.9)	0.2 (0.03–3.6)			
**Time interval T1 – adjuvant therapy onset in mo**							
Mean (SD)	1.0 (0.5)	1.1 (0.8)	1.5 (1.8)		0.479	0.495	0.696
Median (range)	0.8 (0.2–1.9)	1.0 (0.1–3.7)	0.9 (0.4–6.0)				

P-values of statistical tests indicate differences between treatment groups, between irradiated (RT+ ) and non-irradiated (RT-) patients (penultimate column) and between patients with chemotherapy (ChT+) and without chemotherapy (ChT-) (last column), respectively

*SD* standard deviation, *mo* months, *AED* anti-epileptic drug, *T1* baseline neuropsychological assessment, *T2* follow-up neuropsychological assessment

^a^ Extent of resection was defined according to magnetic resonance (MR) or computer tomography (CT) imaging within 21 days post-surgery. Post-surgical residual tumor volume was compared to tumor volume prior to surgery. Gross total resection was defined as no visible residual tumor, subtotal resection as 50–99% excision of tumor volume and partial resection as < 50% excision of tumor volume

^b^ In pilocytic astrocytoma, ependymoma, subependymoma, ganglioglioma

^c^ Comparison of frequencies for extent of resection (gross total resection/subtotal resection/partial resection vs. biopsy)

^d^ Comparison of frequencies for histology (astrocytoma vs. oligodendroglioma)

^e^ Comparison of frequencies for WHO grade (WHO grade 2 vs. WHO grade 3)

^f^ Comparison of frequencies for tumor lateralization (left vs. right)

^g^ Comparison of frequencies for frontal tumor localization (frontal vs. non-frontal)

^h^ Comparison of frequencies for temporal tumor localization (temporal vs. non-temporal)

* *p* < 0.05, ** *p* < 0.01

All patients in this study had undergone either biopsy or partial, subtotal or gross total resection. Extent of resection was defined according to magnetic resonance (MR) or computer tomography (CT) imaging within 21 days post-surgery. Gross total resection was defined as no visible residual tumor, subtotal resection as 50–99% excision of tumor volume and partial resection as < 50% excision of tumor volume. Based on tumor histology, patients were treated either with adjuvant conventional external RT, chemotherapy (ChT) or combined radio-chemotherapy (RChT) according to the German Neuro-Oncology Group (NOA) guidelines and in case of WHO grade 2 or 3 gliomas according to center guidelines, which were subject to change overtime. Since patients within this study were treated prior to publication of the RTOG 98-02-trial [24], many WHO grade 2 glioma patients underwent tumor resection only and did not receive any adjuvant tumor-specific therapy according to a watch-and-wait policy.

### Neurocognitive functioning

In order to assess neurocognitive treatment effects evaluation of (1) psychomotor speed, attention and executive functioning, (2) short-term and working memory, (3) verbal memory and fluency, and (4) figural memory is recommended [25–27]. NeuroCog FX is a computerized neuropsychological test battery comprising eight subtests to assess these neurocognitive functions in a time-saving, reliable, standardized [28, 29] and validated [30] manner. NeuroCog FX meets guidelines [31] such as sensitivity and specificity to detect cognitive deficits in neuro-oncological trials (for details, see Online Resource 1).

### Procedures

Baseline neuropsychological assessment (NPA) was conducted after surgery, before start of adjuvant therapy. Follow-up NPAs were carried out prospectively within regular neurological follow-up consecutively every six months. As we intended to evaluate mid-term treatment-related neurotoxicity, baseline NPA (Timepoint 1, T1) and the latest NPA within a two-year interval after baseline assessment (Timepoint 2, T2, follow-up) were selected for analyses. We aimed to largely exclude a possible impact of tumor progression on results [32–34] by excluding data of patients with confirmed tumor progression within three months after NPA.

### Statistical analyses

To analyze differences with respect to associations with RT and ChT four treatment modalities were distinguished: RT, ChT, RChT and watch-and-wait. In order to increase the size of subgroups, treatment groups were dichotomized for an additional analysis to detect subtle changes in cognitive performance. Accordingly, patients with RT or RChT were categorized as RT+  and patients with ChT only or watch-and-wait as RT-. Correspondingly, patients with ChT or RChT were categorized as ChT+ and patients with RT only or watch-and-wait as ChT-. Two-tailed t-tests for independent samples, one-way analyses of variance (ANOVA), Fisher’s Exact Test and Pearson’s χ^2^ Test were used to test for clinical and sociodemographic differences between groups.

Since NeuroCog FX provides comparative evaluation of individual test scores with respect to normative test scores of an age-adapted cohort of healthy controls, percentile ranks were analyzed as standardized outcome measures [28, 29]. A percentile rank score > 16 and < 84 indicates performance within average of healthy controls.

To evaluate group differences regarding changes of neurocognitive functioning as the primary outcome, multivariate one-way ANOVAs were performed with treatment as fixed factor. Dependent variables included NeuroCog FX percentile ranks which were transformed into difference scores between T2 and T1. A score > 0 indicated improvement and a score < 0 deterioration of performance. To evaluate changes of cognitive functioning within every single patient subgroup, t-tests for dependent samples or repeated measures ANOVAs for percentile ranks were calculated with time of assessment (T1/T2) as within-subject factor and treatment (RT vs. ChT vs. RChT vs. watch-and-wait or RT+ vs. RT- or ChT+ vs. ChT-) as between-subject factor. The impact of extent of resection and histopathological characteristics on cognitive functioning were referred to as secondary outcomes. Analyses were performed in IBM SPSS Statistics 25 with a significance level of 0.05.

## Results

### Sociodemographic and clinical characteristics

Of 280 patients initially included in this project, 180 patients were excluded from analyses because of missing NPA at any timepoint, tumor recurrence before T2, extended time intervals between surgery and baseline NPA, because they were lost to follow-up or refused to complete NPA (n = 14). Eighty-six patients with histopathologic diagnosis of glioma were analyzed. At the time patients had been recruited their tumors were classified according to the WHO classification system 2007, i.e. 48 patients had WHO grade 2 gliomas (“LGG”), 27 WHO grade 3 gliomas and 11 WHO grade 4 gliomas. Since we aimed to align the analyses as best as possible to the current tumor classification system, all available tumor samples were re-classified according to the WHO classification of 2021 [1]. Subsequently, the tumor cohort included 33 astrocytomas, IDH-mutant, 18 oligodendrogliomas, IDH-mutant and 1p/19q-codeleted, two glioblastomas, IDH-wildtype, eight other entities (e.g. ependymoma, pilocytic astrocytoma) and 25 gliomas that could not be re-evaluated due to lack of available tumor tissue (Table 1).

The extent of primary resection was gross total in 34 out of 86 patients, subtotal in 23, partial in 13 and biopsy in 16 patients (Table 1). In 61 patients post-surgery MR imaging scans and in 25 patients post-surgery CT scans were used to evaluate extent of resection.

Ten out of 86 patients (12%) received conventional external focal RT alone, 21 (24%) chemotherapy alone (ChT; 16 (76%) temozolomide, 5 (24%) PCV or nitrosourea), 24 (28%) were treated with radio-chemotherapy (RChT; concomitant: 16 (67%) temozolomide; 8 (33%) PCV or nitrosourea) and 31 (36%) had been followed by watch-and-wait strategy after surgery. RT+  patients received fraction doses of 1.8–2.0 Gy, with a median total dose of 59.4 (range 39.6–60.0) Gy. One patient received fraction doses > 2.0 Gy. One RChT patient received WBRT and one RT patient received interstitial RT (i.e. radioactive material is directly placed into or close to the tumor).

Baseline NPA was largely conducted within one week (median 7 days, range one day to 15 weeks) after surgery and before start of adjuvant therapy in all cases. No correlation between neurocognitive function and length of time interval between surgery and NPA (3–13 days) was found previously [35]. Patients underwent serial NPA every six months. In all RT+  patients, follow-up NPA exceeded a six months interval after completion of RT to rule out acute or early delayed radiation toxicity. Thus, median T1-T2 interval was 16.8 (range 5.9–31.1) months.

### Change of neurocognitive functioning within two years after surgery

**Fig. 1:**
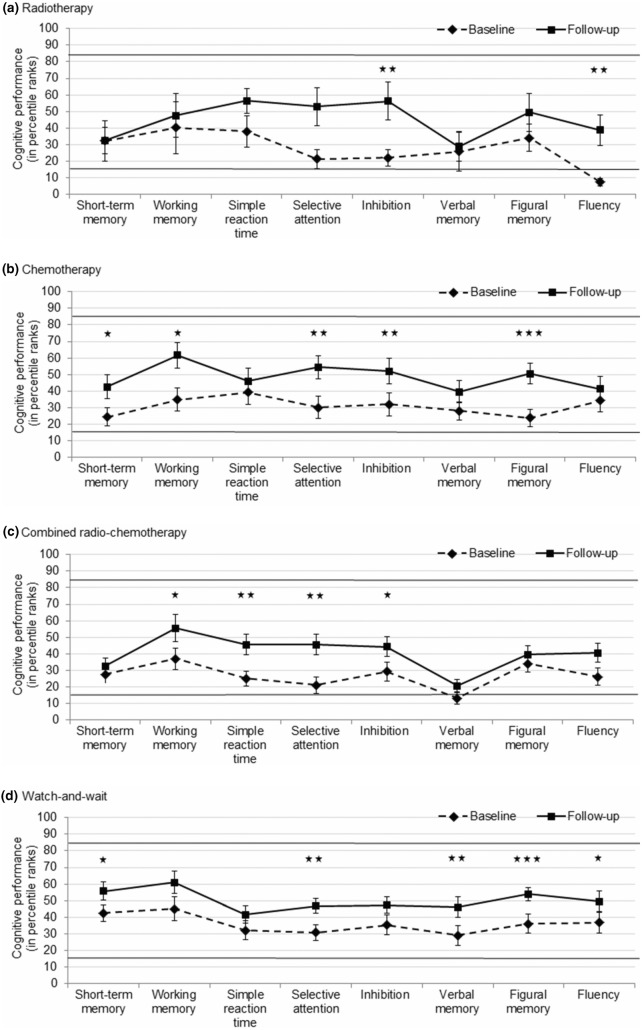
Cognitive performance (in percentile ranks) in NeuroCog FX subtests at baseline and follow-up, separated for treatment groups. **a** Radiotherapy (n = 10). **b** Chemotherapy (n = 21). **c** Combined radio-chemotherapy (n = 24). **d** Watch-and-wait (n = 31). Asterisks indicate statistically significant changes (i.e. improvement) in cognitive performance; * *p* < 0.05, ** *p* < 0.01, *** *p* < 0.001; bars indicate standard error of mean

With the exception of two mean scores at T1 (fluency in RT patients and verbal memory in RChT patients), mean cognitive performance of all treatment groups was categorized within average of healthy controls (percentile ranks between 16 and 84) in all domains at T1 and T2. All treatment groups numerically and often even significantly improved in all cognitive domains (Fig. 1). ANOVAs revealed that the extent of change in cognitive performance did not differ between treatment groups in any cognitive domain (all *p*s ≥ 0.076; additional data are given in Online Resource 2).

Regarding performance at T2, analyses indicated diverging performance levels in short-term memory (*p* = 0.022) and verbal memory (*p* = 0.013) between groups. Pairwise post-hoc comparisons indicated that cognitive performance of watch-and-wait patients at T2 was better than that of RChT patients with respect to short-term memory (mean score 55.7 [standard deviation (SD) 31.1] vs. 32.5 [SD 24.1], *p* = 0.028; mean difference 23.2, 95% confidence interval (CI) 7.8–38.6) and verbal memory (mean score 45.9 [SD 34.4] vs. 20.1 [SD 17.7], *p* = 0.011; mean difference 25.8, 95% CI 11.4–40.3), despite equivalent baseline performance levels (not shown).

### Change of neurocognitive functioning associated with radiotherapy

**Fig. 2:**
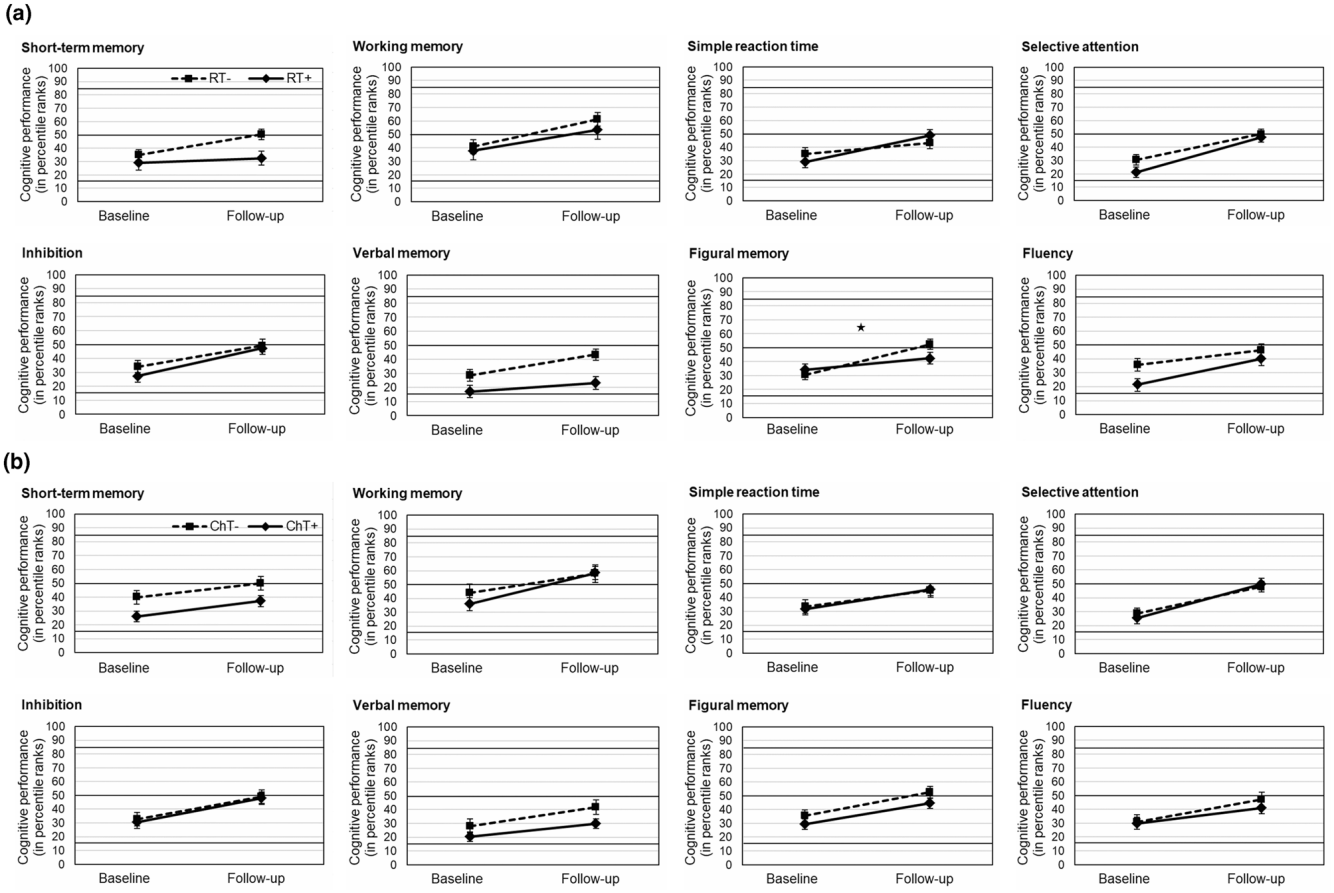
Change of cognitive functioning within a two-year follow-up. Graphs indicate mean cognitive performance (in percentile ranks) in NeuroCog FX subtests at baseline after surgery and at two-year follow-up, separated for dichotomized treatment groups. The asterisk indicates a statistically significant difference in cognitive change between groups; * *p* < 0.05; bars indicate standard error of mean. **a** Irradiated patients (RT+, radiotherapy only or combined radio-chemotherapy, n = 34) and non-irradiated patients (RT-, chemotherapy only or watch-and-wait, n = 52). **b** Patients with chemotherapy (ChT+, chemotherapy only or combined radio-chemotherapy, n = 45) and without chemotherapy (ChT-, radiotherapy only or watch-and-wait, n = 41)

When comparing cognitive performance of RT+  (n = 34) and RT- (n = 52) patients, repeated measures ANOVA revealed a significant interaction of radiotherapy and timepoint (i.e. a group difference in longitudinal change) with respect to figural memory (*p* = 0.029; mean difference 13.5, 95% CI 1.4–25.7) only. RT- patients improved from initial percentile rank 30.6 (SD 26.2) up to 52.4 (SD 23.0), whereas RT+  patients improved from initial percentile rank 34.1 (SD 24.7) up to 42.4 (SD 29.1) at follow-up. Similarly, RT- patients showed a numerically stronger increase of performance than RT+  patients in short-term memory (mean score 15.2 [SD 28.9] vs. 3.6 [SD 28.9], *p* = 0.071; mean difference 11.6, 95% CI -1.0–24.3). By contrast, RT+  patients showed a numerically stronger improvement of performance than RT- patients in reaction time (mean score 20.0 [SD 27.2] vs. 8.3 [SD 32.1], *p* = 0.088; mean difference 11.6, 95% CI 1.8–25.0) (Table 2 and Fig. 2a). No differences with respect to radiation occurred for working memory, selective attention, inhibition, verbal memory and fluency (all *p*s ≥ 0.125). RT- patients had higher overall performance levels than RT+  patients in short-term memory (mean score 42.7 [SD 26.9] vs. 30.8 [SD 23.2], *p* = 0.037; mean difference 12.0, 95% CI 0.8–23.2) and verbal memory (mean score 36.0 [SD 27.7] vs. 20.1 [SD 21.6], *p* = 0.007; mean difference 15.9, 95% CI 4.4–27.3), when considering cognitive performance averaged for T1 and T2. Additional data on an individual patient level are given in Online Resource 3.

**Table 2:** Extent of change in cognitive performance between baseline and follow-up neuropsychological assessment represented by mean differences (M_diff_) of percentile ranks (T2-T1; a score > 0 indicates a numerical improvement of cognitive performance over time), separated for dichotomized treatment groups (without radiotherapy [RT-] vs. with radiotherapy [RT+] and without chemotherapy [ChT-] vs. with chemotherapy [ChT+])

	RT- (n = 52)	RT+ (n = 34)	ANOVA			ChT- (n = 41)	ChT+ (n = 45)	ANOVA		
	M_diff_ (95% CI)	M_diff_ (95% CI)	F-value	df	p-value	M_diff_ (95% CI)	M_diff_ (95% CI)	F-value	df	p-value
Short-term memory	15.2 (7.2–23.3)	3.6 (-6.5–13.7)	3.34	1,84	0.071	10.1 (0.2–20.1)	11.1 (2.8–19.3)	0.02	1,84	0.885
Working memory	20.3 (6.4–34.2)	15.5 (2.5–28.4)	0.23	1,83	0.630	14.1 (-1.2–29.4)	22.3 (9.5–35.1)	0.71	1,83	0.403
Simple reaction time	8.3 (-0.6–17.3)	20.0 (10.3–29.6)	2.98	1,83	0.088	11.6 (1.3–22.0)	14.0 (5.2–22.8)	0.13	1,83	0.724
Selective attention	19.3 (11.3–27.3)	26.4 (13.5–39.3)	1.00	1,81	0.320	19.1 (9.1–29.1)	24.5 (14.8–34.1)	0.60	1,81	0.440
Inhibition	15.2 (6.2–24.2)	19.9 (7.6–32.2)	0.41	1,82	0.523	16.5 (5.1–27.8)	17.4 (8.0–26.8)	0.02	1,82	0.897
Verbal memory	14.8 (7.0–22.5)	6.1 (-0.8–13.0)	2.40	1,82	0.125	13.6 (5.1–22.0)	9.4 (2.1–16.7)	0.58	1,82	0.449
Figural memory	21.8 (14.8–28.9)	8.3 (-2.4–19.0)	4.95	1,76	0.029* ^a^	17.1 (7.2–26.9)	15.1 (6.9–23.2)	0.10	1,76	0.752
Fluency	10.4 (1.6–19.1)	18.6 (6.7–30.5)	1.31	1,82	0.256	16.5 (6.3–26.7)	11.0 (1.1–20.8)	0.61	1,82	0.438

ANOVA test statistics illustrate differences in extent of cognitive change between RT- patients and RT+  patients and between ChT- patients and ChT+ patients, respectively. Asterisks indicate statistically significant group differences

*CI* confidence interval, *df* degree of freedom

^a^ Non-irradiated patients show a significantly greater increase in figural memory performance than irradiated patients within two years after post-surgery baseline assessment

* *p* < 0.05

### Change of neurocognitive functioning associated with chemotherapy

ChT+ (n = 45) and ChT- (n = 41) patients did not differ in any cognitive domain with respect to longitudinal increase or decrease of performance (all *p*s ≥ 0.403). No tendency for any difference in cognitive change between groups occurred (Table 2 and Fig. 2b). However, ChT- patients showed a higher overall performance level than ChT+ patients in short-term memory (mean score 45.0 [SD 26.9] vs. 31.6 [SD 23.8], *p* = 0.017; mean difference 13.4, 95% CI 2.5–24.3), when considering cognitive performance averaged for T1 and T2.

### Neurocognitive functioning related to tumor and surgery characteristics

Comparison of patients with oligodendroglioma (n = 11 WHO grade 2 and n = 7 WHO grade 3) and astrocytoma (n = 17 WHO grade 2 and n = 11 WHO grade 3) revealed that at T1 and T2, there was no difference between groups in any cognitive domain (all *p*s ≥ 0.071). Both groups improved numerically in all cognitive domains. While patients with oligodendroglioma improved significantly in reaction time, selective attention, inhibition and verbal memory (*p*s ≤ 0.031), significant improvements in verbal memory, figural memory and selective attention (*p*s ≤ 0.044) were seen in astrocytoma. A stronger improvement concerning inhibition was seen in oligodendroglioma as compared to astrocytoma (mean score 26.7 [SD 28.2] vs. 6.4 [SD 31.8], *p* = 0.034; mean difference 20.3, 95% CI 1.6–38.9). Additional data are given in Online Resource 4.

In astrocytoma, irradiation and chemotherapy had no diverging effects on cognitive functions when comparing the extent of cognitive change in RT+  and RT- patients and ChT+ and ChT- patients, respectively (all *p*s ≥ 0.150, not shown). The same was true for irradiation in oligodendroglioma. However, comparison of ChT+ and ChT- in oligodendroglioma patients showed a stronger improvement of ChT- patients in reaction time (mean score 45.6 [SD 30.9] vs. 8.5 [SD 29.9], *p* = 0.033; mean difference 37.1, 95% CI 3.5–70.8, not shown). Altogether, in both glioma groups, RT- patients improved in more cognitive domains than RT+  patients and ChT+ patients improved in more domains than ChT- patients (for details, see Online Resource 5). Non-irradiated oligodendroglioma patients showed a stronger improvement than non-irradiated astrocytoma patients concerning inhibition (mean score 31.4 [SD 27.5] vs. 2.1 [SD 25.1], *p* = 0.004; mean difference 29.2, 95% CI 9.9–48.6). Similarly, oligodendroglioma patients without chemotherapy showed a stronger improvement than astrocytoma patients without chemotherapy concerning reaction time (mean score 45.6 [SD 30.9] vs. 2.5 [SD 32.4], *p* = 0.019; mean difference 43.1, 95% CI 7.9–78.3) and inhibition (mean score 42.2 [SD 31.1] vs. 5.6 [SD 31.3], *p* = 0.041; mean difference 36.6, 95% CI 1.7–71.5) (not shown).

At T1, patients with gross total, subtotal or partial resection (n = 70) showed numerically worse performance than patients with biopsy (n = 16) in most domains (except verbal memory and fluency) with significantly lower performance in working memory (mean score 35.9 [SD 35.4] vs. 55.9 [SD 37.3], *p* = 0.047; mean difference 20.0, 95% CI 0.3–39.7) and reaction time (mean score 28.8 [SD 26.9] vs. 49.2 [SD 36.3], *p* = 0.013; mean difference 20.4, 95% CI 4.5–36.3). Resected patients improved in all cognitive domains (all *p*s ≤ 0.001), whereas patients with biopsy improved in selective attention (*p* = 0.019; mean difference 21.5, 95% CI 4.2–38.8) and figural memory (*p* = 0.048; mean difference 13.6, 95% CI 0.1–27.1) only (for details, see Online Resource 6). At T2, cognitive performance of groups predominantly converged.

## Discussion

The present study aimed to address the “mid-term” treatment-related effects of RT, ChT, RChT and watch-and-wait on cognitive functioning (i.e. the primary outcome) of WHO grade 1–4 glioma patients treated between 2004 and 2012. The aspect of clinical significance was addressed by using standardized outcome measures (i.e. percentile ranks) that form the basis for evaluating a deviation from “healthy” functionality.

The four treatment groups showed no differences in cognitive change two years after first-line surgery; moreover, they showed numerical and often even statistically significant improvements of cognitive functioning when comparing baseline and follow-up; this effect was more pronounced in patients with gross total, subtotal or partial resection than in those with biopsy.

Improvements of cognitive performance in the present study may just represent an effect of recovery from surgery, but may as well reflect “consolidation” of compromised brain function after elimination of glioma cells, networks and their detrimental metabolic and (patho)neurophysiological effects on normal surrounding tissue [36–39]. In this vein functional connectivity is disturbed by glioma even in brain tissue remote from the visible tumor, subsequently affecting cognitive performance [40]. In patients having undergone biopsy only and thus not having experienced reduction of their tumor volume at baseline significant improvements were observed in two cognitive domains only, whereas patients with gross total, subtotal or partial resection showed significant improvements in all cognitive domains. One possible explanation may be that biopsies represent the preferred method for large and/or deeply located tumors [11] with worse prognosis in terms of cognitive decline over time and do not result in volume reduction or tumor network disruption. In contrast, resections apply to accessible gliomas, effectively reduce tumor volume and disrupt tumor networks but are more invasive and potentially harmful, such that they carry a higher risk of immediate neurologic deterioration, and a more compromised state to recover from in the following (months). It is of note that immediate (“baseline”) NPA showed numerically or significantly worse performance in resected patients in most cognitive domains, such that a sustained effect on recovery of brain function, i.e. neurocognition was detectable at T2. Admittedly, there was a significant difference between resected patients and patients with biopsy concerning the numerical distribution of adjuvant treatment modalities that might have confounded results at T2. However, due to the small sample size of these subgroups it is impossible to draw firm conclusions from this observation. Consistent with the finding of more extensive improvements in resected patients in our study, a moderate correlation of disturbed functional connectivity in the lesional hemisphere with tumor volume – though no correlation was found for tumor volume and cognitive performance – and a strong influence of the main tumor on cognitive functions were suggested [40].

In oligodendroglioma patients we found a greater potential to improve in attentional and executive functions than in astrocytoma patients, especially in patients followed by a watch-and-wait strategy, although impairments in these cognitive domains often persist after the postoperative recovery period in brain tumor patients in general [41]. A greater potential to improve was seen in oligodendroglioma than in astrocytoma patients with a better outcome in RT- and ChT+ patients in both groups. This underlines the favorable disposition of oligodendroglioma in neurocognitive outcome as compared to astrocytoma. However, the favorable neurocognitive outcome in oligodendroglioma patients may not purely reflect a result of tumor biology within this entity but may as well be a consequence of the treatment modality (i.e. chemotherapy) which was applied in 50% of oligodendrogliomas and in only 21.4% of astrocytomas in our series. In contrast, combined radio-chemotherapy was applied in 28.6% of astrocytoma patients, whereas only 22.2% of oligodendroglioma patients received this treatment in our series. Therefore, the uneven distribution of treatment modalities in astrocytoma and oligodendroglioma patients may have confounded results.

It is of note that no advantage of irradiation or chemotherapy was seen in astrocytoma patients, though astrocytomas are capable to build widely distributed tumor cell networks that disrupt neuronal networks and functional connectivity and compromise cognitive functions [40]. In this regard, our study could not support the assumption that radiation or chemotherapy suppress this network-effect and thus contribute to an improvement of cognitive functioning.

ChT+ patients showed no significant cognitive deterioration as compared to ChT- patients. We assume that chemotherapy did not induce mid-term neurotoxicity in our sample in line with a favorable neurological side effect profile of temozolomide [42, 43]. The present results match with previous evidence on marginal [44] or even no adverse impact of chemotherapy on cognitive functioning within 12 months following RChT in “high-grade glioma” (HGG) [45]. In “HGG” patients, who were assessed before, during and after standard RChT, cognitive functioning remained stable or improved in 70% of the patients [46]. Likewise, RTOG 05-25-trial showed no differences in neurocognitive functioning between dose-intensifying temozolomide versus standard chemo-radiotherapy in newly diagnosed glioblastoma within six months after chemotherapy [19].

It is assumed that RT may negatively affect health-related QoL through irreversible brain damage resulting in cognitive deficits, which was true in former analyses evaluating radiation sequelae of “historic” regimens and methodology [5, 12, 47]. In the present study no deteriorating effect of RT was found two years following focal RT with mean total doses of 59.4 Gy (fraction dose 1.8–2.0 Gy). Similarly, several studies reported no significant long-term cognitive impairments following focal radiation with total doses of 50.4–64.8 Gy and fraction doses of 1.8 Gy [48–50]. When comparing cognitive performance between dichotomized groups of RT+  and RT- patients, however, a significantly lower improvement (nevertheless an improvement) of figural memory was detected in RT+  patients in the present study. Thus, it cannot be ruled out that exposure to radiation may delay or counteract postoperative recovery of memory functions. By contrast, attention and executive functions seem to recover within two years after surgery irrespective of the application of irradiation. One possible explanation may be that hippocampal structures with neuronal progenitor and stem cell compartments - regarded as being essential for memory - are particularly sensitive to RT due to its adverse impact on hippocampal neurogenesis and myelin production [51, 52].

Since Douw and colleagues reported significant increases of attentional deficits that were independent of e.g. fraction dose, extent of resection and AED approximately 12 years following RT [12] cognitive deficits may become apparent after several years only. Thus, a long-term follow-up of our patients is required.

Though patients within this series had been recruited between 2004 and 2012 such that their gliomas had been classified according to the previous WHO classification systems published in 2000 and 2007, the primary endpoint of this trial “mid-term neurocognitive function” obviously is not affected by modification of histopathological and molecular classification. However, to further analyze subgroups of distinct gliomas in association with effects of different treatment modalities, all available tumor material had been re-assessed for molecular markers determining the current classification system. It is of note that five out of 11 glioblastomas were re-classified as astrocytomas, IDH-mutant WHO grade 4, i.e. gliomas with a different biology. Thus, it cannot be completely ruled out that tumor characteristics confounded results of overall analysis of neurocognition.

The present study is not without limitations. Since we had included patients treated in large university centers we cannot rule out that our cohort represents a highly selected population, which might have biased results. Furthermore, the rather small sample size and partly heterogeneous patient population with small subsamples may have limited statistical power and hence obscured the demonstration of statistically significant differences concerning treatment modalities. We increased the size of subgroups by dichotomizing treatment groups in order to detect subtle changes of cognitive performance, while we appreciate that these composite groups do not allow to draw firm conclusions concerning the mere effect of irradiation and chemotherapy on neurocognition. As we have prospectively observed a patient group and evaluated cognitive functioning at different timepoints for several years (i.e. between 2004 and 2012), a change in established treatments and imaging techniques of brain tumors unfortunately could not be avoided or controlled for. Therefore, we cannot rule out the possibility that the present results on cognitive functioning are confounded by classification systems and treatment protocols that are outdated. Furthermore, in 25 out of 86 patients a CT was used to evaluate extent of resection instead of MR imaging, which is not state of the art. In addition, the evaluation of cognitive change using the reliable change index would have been a reasonable alternative to evaluate change in cognitive performance over time. However, since the focus was on the magnitude of cognitive change rather than the mere analyses of deterioration or improvement and to avoid any loss of information, percentile ranks were used instead. Furthermore, NeuroCog FX is standardized [28, 29] and validated [30] but one may argue, that the mere absence of cognitive deficits may be due to shortcomings of this test, anyway. The number of patients with cognitive decline may be underestimated since patients who refused follow-up assessment might be in clinically worse conditions, i.e. positive selection of patients. Moreover, test repetitions may in part be accountable for cognitive improvements. However, since the interval between two NPAs was at least six months and parallel versions of subtests were used it is unlikely that transfer or practice effects confounded the present results. Principally it has to be considered that the mere lack of significant differences in primary and secondary outcome measures in this rather small and heterogeneous sample does not exclude the presence of subtle changes that may be significant in larger populations.

In sum, the present study suggests no relevant changes of cognitive performance in dependence of treatment in WHO grade 1–4 glioma patients in a mid-term follow-up.

## Supplementary Information

Below is the link to the electronic supplementary material.

Supplementary file1 (PDF 381 KB)

Supplementary file2 (PDF 437 KB)

Supplementary file3 (PDF 399 KB)

Supplementary file4 (PDF 441 KB)

Supplementary file5 (PDF 819 KB)

Supplementary file6 (PDF 508 KB)

## Data Availability

The datasets generated or analyzed during this study are available from the corresponding author on reasonable request.
