# Supplementary material for: Mid-term treatment-related cognitive sequelae in glioma patients
Source: J Neurooncol. 2022 Jul 7;159(1):65–79. doi: 10.1007/s11060-022-04044-1 (PMC9325813; doi:10.1007/s11060-022-04044-1)
Supplement: Supplementary file 1 — Supplementary file1 (PDF 381 KB) [file 11060_2022_4044_MOESM1_ESM.pdf]

Supplementary material (Online Resource 1)

Manuscript title:	Mid-term treatment-related cognitive sequelae in glioma patients
Journal:	Journal of Neuro-Oncology
Authors:	Sabine Schlömer, Jörg Felsberg, Milena Pertz, Bettina Hentschel, Markus Löffler, Gabriele Schackert, Dietmar Krex, Tareq Juratli, Joerg Christian Tonn, Oliver Schnell, Hartmut Vatter, Matthias Simon, Manfred Westphal, Tobias Martens, Michael Sabel, Martin Bendszus, Nils Dörner, Klaus Fliessbach, Christian Hoppe, Guido Reifenberger, Michael Weller, Uwe Schlegel
Corresponding author:	Sabine Schlömer
Affiliation of corresponding author:	Department of Neurology, University Hospital Knappschaftskrankenhaus, Ruhr University Bochum,<br>In der Schornau 23-25, D-44892 Bochum, Germany
E-Mail address of corresponding author:	sabine.schloemer@kk-bochum.de

Description of the German Glioma Network and NeuroCog FX

The German Glioma Network

The German Glioma Network (GGN) is a prospective, non-interventional cohort study involving eight clinical centers at university hospitals in Germany (www.gliomnetzwerk.de), and was supported by the German Cancer Aid for three funding periods from 2004 to 2012. The GGN realizes a collaboration of neurological, neurosurgical and neuropathological expertise dealing with translational research projects and issues of optimal treatment, prognostic factors and molecular analyses in glioma. The GGN provides an ideal and essential infrastructure to evaluate long-term prospective treatment-related neurotoxicity in a large patient cohort.

NeuroCog FX

Table ESM-1 NeuroCog FX: Description of cognitive domains, tasks and standardized performance scores of NeuroCog FX subtests [28, 29, 30]

Subtest	Domain	Task description	Norms for performance scores
Digit Span	Verbal short-term memory	Presentation of a series of digits; immediate reproduction with digit keys. Increasing difficulty after correct answers.	Number of correct reproductions
Two back-test	Working memory	Continuous presentation of single digits. Key press (space bar), when the present digit is identical with the penultimate digit.	Number of correct reactions minus number of incorrect reactions
Simple reaction time	Reaction time, alertness	Reaction at the appearance of a blue circle (space bar).	Median of reaction times
Selective attention (go/nogo)	Selective attention, inhibition of irrelevant reactions	Reaction at the appearance of a blue circle (space bar), ignoration of yellow circles.	Median of reaction times
Inhibition (go/nogo)	Inhibition of irrelevant reactions, cognitive flexibility	Changed rule: Reaction at the appearance of yellow circles (space bar), ignoration of blue circles.	Median of reaction times
Verbal memory	Verbal memory: verbal learning and recognition	Word list learning: 3 learning trials, 12 words. Yes/No-recognition (test items : distractor items 1 : 2) after each learning trial and delayed recognition after subtest „figural memory“. Yes = space bar (reaction time interval: 2 seconds).	Number of correctly recognized test items (hits) minus number of incorrect "Yes"-classifications (false alarms)
Figural memory	Figural memory: figural (non-verbal) learning and recognition	Analogous to verbal memory subtest. 3 learning trials, 8 checker board-like pattern with 4 highlighted fields in a 3x3 matrix.	Analogous to verbal memory subtest
Verbal fluency	Phonematic-literal word fluency	Naming as many words beginning with letter "P" ("L", "S" in alternative versions)	Number of correctly produced words
