# Supplementary material for: Mid-term treatment-related cognitive sequelae in glioma patients
Source: J Neurooncol. 2022 Jul 7;159(1):65–79. doi: 10.1007/s11060-022-04044-1 (PMC9325813; doi:10.1007/s11060-022-04044-1)
Supplement: Supplementary file 2 — Supplementary file2 (PDF 437 KB) [file 11060_2022_4044_MOESM2_ESM.pdf]

Supplementary material (Online Resource 2)

Manuscript title:	Mid-term treatment-related cognitive sequelae in glioma patients
Journal:	Journal of Neuro-Oncology
Authors:	Sabine Schlömer, Jörg Felsberg, Milena Pertz, Bettina Hentschel, Markus Löffler, Gabriele Schackert, Dietmar Krex, Tareq Juratli, Joerg Christian Tonn, Oliver Schnell, Hartmut Vatter, Matthias Simon, Manfred Westphal, Tobias Martens, Michael Sabel, Martin Bendszus, Nils Dörner, Klaus Fliessbach, Christian Hoppe, Guido Reifenberger, Michael Weller, Uwe Schlegel
Corresponding author:	Sabine Schlömer
Affiliation of corresponding author:	Department of Neurology, University Hospital Knappschaftskrankenhaus, Ruhr University Bochum, In der Schornau 23-25, D-44892 Bochum, Germany
E-Mail address of corresponding author:	sabine.schloemer@kk-bochum.de

Table ESM-2 Neurocognitive change within and between treatment groups. T-test statistics for mean extent of cognitive improvement (i.e. mean difference [in percentile ranks] between baseline [T1] and follow-up [T2] neuropsychological assessment), separated for patients with radiotherapy (RT), chemotherapy (ChT), combined radio-chemotherapy (RChT) and patients followed by watch-and-wait strategy. Asterisks indicate statistically significant cognitive improvement. ANOVA (with F-statistics) for differences in cognitive change between treatment groups. CI confidence interval, df degree of freedom. * $p < .05$, ** $p < .01$, *** $p < .001$

	**RT (n = 10)**			**ChT (n = 21)**			**RChT (n = 24)**			**Watch-and-wait (n = 31)**			**ANOVA**		
	Mean improvement between T1 and T2 (95% CI) [in percentile ranks]	t-value (df)	p-value	Mean improvement between T1 and T2 (95% CI) [in percentile ranks]	t-value (df)	p-value	Mean improvement between T1 and T2 (95% CI) [in percentile ranks]	t-value (df)	p-value	Mean improvement between T1 and T2 (95% CI) [in percentile ranks]	t-value (df)	p-value	F-value	df	p-value
Short-term memory	0.4 (-26.9-27.7)	0.03 (9)	.974	18.1 (4.9-31.3)	2.87 (20)	.010*	4.9 (-5.6-15.4)	0.97 (23)	.344	13.3 (2.6-24.0)	2.54 (30)	.017*	1.26	3,82	.293
Working memory	7.3 (-18.7-33.3)	0.65 (8)	.534	26.7 (4.8-48.5)	2.55 (20)	.019*	18.5 (2.6-34.4)	2.41 (23)	.025*	16.0 (-2.9-35.0)	1.73 (30)	.094	0.44	3,81	.727
Simple reaction time	18.5 (-1.9-38.9)	2.05 (9)	.07	6.76 (-6.6-20.2)	1.05 (20)	.305	20.6 (8.9-32.4)	3.64 (22)	.001**	9.4 (-3.1-22.0)	1.53 (30)	.136	1.01	3,81	.392
Selective attention	31.6 (-3.8-67.0)	2.11 (7)	.072	24.3 (10.3-38.4)	3.61 (20)	.002**	24.6 (10.2-38.9)	3.54 (22)	.002**	15.9 (5.9-25.8)	3.26 (30)	.003**	0.73	3,79	.535
Inhibition	34.3 (11.8-56.7)	3.61 (7)	.009**	20.0 (8.0-32.0)	3.47 (20)	.002**	15.1 (0.2-30.0)	2.10 (23)	.047*	11.9 (-1.3-25.0)	1.85 (30)	.075	1.07	3,80	.365
Verbal memory	3.1 (-12.5-18.7)	0.45 (9)	.663	11.5 (-1.5-24.4)	1.85 (20)	.080	7.4 (-0.7-15.5)	1.91 (21)	.071	17.0 (6.8-27.1)	3.41 (30)	.002**	1.06	3,80	.373
Figural memory	15.3 (-15.0-45.6)	1.14 (9)	.283	26.7 (15.0-38.4)	4.79 (19)	<.001***	5.4 (-5.1-15.9)	1.06 (23)	.302	17.8 (8.9-26.7)	4.12 (23)	<.001***	2.39	3,74	.076
Fluency	31.4 (11.3-51.4)	3.70 (7)	.008**	7.1 (-6.8-21.0)	1.07 (20)	.300	14.4 (-0.4-29.1)	2.02 (23)	.055	12.6 (0.8-24.5)	2.17 (30)	.038*	1.13	3,80	.344
