# Supplementary material for: Mid-term treatment-related cognitive sequelae in glioma patients
Source: J Neurooncol. 2022 Jul 7;159(1):65–79. doi: 10.1007/s11060-022-04044-1 (PMC9325813; doi:10.1007/s11060-022-04044-1)
Supplement: Supplementary file 3 — Supplementary file3 (PDF 399 KB) [file 11060_2022_4044_MOESM3_ESM.pdf]

Supplementary material (Online Resource 3)

Manuscript title:	Mid-term treatment-related cognitive sequelae in glioma patients
Journal:	Journal of Neuro-Oncology

Authors:	Sabine Schlömer, Jörg Felsberg, Milena Pertz, Bettina Hentschel, Markus Löffler, Gabriele Schackert, Dietmar Krex, Tareq Juratli, Joerg Christian Tonn, Oliver Schnell, Hartmut Vatter, Matthias Simon, Manfred Westphal, Tobias Martens, Michael Sabel, Martin Bendszus, Nils Dörner, Klaus Fliessbach, Christian Hoppe, Guido Reifenberger, Michael Weller, Uwe Schlegel
Corresponding author:	Sabine Schlömer
Affiliation of corresponding author:	Department of Neurology, University Hospital Knappschaftskrankenhaus, Ruhr University Bochum, In der Schornau 23-25, D-44892 Bochum, Germany
E-Mail address of corresponding author:	sabine.schloemer@kk-bochum.de

Change of cognitive performance on individual patient level within two years after surgery

As group mean values may mask individual cognitive dysfunction, performance of individual patients was analyzed according to defined cut-off-values (described previously [28, 30]). It was intended to identify individual, clinically relevant cognitive dysfunction and its change over time. For each cognitive domain and every single patient, presence of cognitive dysfunction was evaluated for baseline (for no cognitive dysfunction at baseline see Table ESM-3a and for cognitive dysfunction at baseline see Table ESM-3b) and follow-up assessment. Individual percentile rank scales were transformed into dichotomous variables (impaired vs. not impaired). For this purpose, published cut-off values to define a cognitive deficit [28, 30] were used. After that, the course of performance was analyzed and either an improvement, a decline or a stable performance were rated. Between treatment groups, odds ratios (OR) were calculated to indicate the relation of relative frequencies.

Odds ratios with respect to a deterioration or improvement showed no difference between RT+ patients and RT- patients in any cognitive domain. However, odds ratios supposing to indicate a deterioration in figural memory tended to show a difference between groups: for figural memory 81% of RT+ patients showed stable performance, yet 19% deteriorated. 98% of RT- patients showed stable performance whereas only 2% declined, with OR = 13.57 [CI 95%: 0.7, 262.23]. Odds ratios with respect to a deterioration or an improvement showed no difference between ChT+ and ChT- patients in any cognitive domain.

Table ESM-3a Percentage (n) of non-irradiated (RT-) / irradiated (RT+) patients and patients without (ChT-) / with (ChT+) chemotherapy showing stable performance or cognitive decline after having been diagnosed with no cognitive dysfunction at baseline. Odds ratios (OR) and confidence intervals (CI) for percentile ranks are shown

Patients without cognitive dysfunction[a] at baseline										
	RT-		RT+		OR (95% CI)	ChT-		ChT+		OR (95% CI)
Cognitive domain	stable	declined	stable	declined		stable	declined	stable	declined	
Short-term memory[b]	90 (26)	10 (3)	86 (12)	14 (2)	1.44 [0.21;9.81]	83 (20)	17 (4)	95 (18)	5 (1)	0.28 [0.03;2.72]
Working memory[c]	91 (31)	9 (3)	84 (16)	16 (3)	1.94 [0.35;10.72]	92 (22)	8 (2)	86 (25)	14 (4)	1.76 [0.29;10.56]
Simple reaction time[d]	94 (32)	6 (2)	96 (24)	4 (1)	0.67 [0.06;7.79]	96 (25)	4 (1)	94 (31)	6 (2)	1.61 [0.14;18.83]
Selective attention[d]	94 (34)	6 (2)	84 (16)	16 (3)	3.19 [0.48;21]	89 (24)	11 (3)	93 (26)	7 (2)	0.62 [0.09;4.01]
Inhibition[d]	92 (33)	8 (3)	85 (17)	15 (3)	1.94 [0.35;10.67]	93 (26)	7 (2)	86 (24)	14 (4)	2.17 [0.36;12.92]
Verbal memory[d]	90 (26)	10 (3)	69 (9)	31 (4)	3.85 [0.72;20.62]	86 (18)	14 (3)	81 (17)	19 (4)	1.41 [0.27;7.26]
Figural memory[d]	98 (29)	2 (0)	81 (21)	19 (5)	13.57 [0.7;262.23]	88 (22)	12 (3)	93 (28)	7 (2)	0.52 [0.08;3.41]
Fluency[c]	84 (27)	16 (5)	83 (10)	17 (2)	1.08 [0.18;6.49]	84 (16)	16 (3)	84 (21)	16 (4)	1.02 [0.2;5.2]

[a] Cognitive dysfunction according to cut-off-values [28, 30] defined as standard value ≤ 93, standard value ≤ 88, standard value ≤ 85 respective percentile rank ≤ 20, percentile rank ≤ 16, percentile rank ≤ 10
[b] Cognitive dysfunction defined as standard value ≤ 93 respective percentile rank ≤ 20
[c] Cognitive dysfunction defined as standard value ≤ 88 respective percentile rank ≤ 16
[d] Cognitive dysfunction defined as standard value ≤ 85 respective percentile rank ≤ 10

Table ESM-3b Percentage (n) of non-irradiated (RT-) / irradiated (RT+) patients and patients without (ChT-) / with (ChT+) chemotherapy showing stable performance or cognitive improvement after having been diagnosed with cognitive dysfunction at baseline. Odds ratios (OR) and confidence intervals (CI) for percentile ranks are shown

Patients with cognitive dysfunction[a] at baseline

	**RT-**		**RT+**		**OR (95% CI)**	**ChT-**		**ChT+**		**OR (95% CI)**
Cognitive domain	stable	improved	stable	improved		stable	improved	stable	improved	
Short-term memory[b]	48 (11)	52 (12)	60 (12)	40 (8)	0.61 [0.18;2.05]	47 (8)	53 (9)	58 (15)	42 (11)	0.65 [0.19;2.23]
Working memory[c]	28 (5)	72 (13)	21 (3)	79 (11)	1.41 [0.27;7.28]	38 (6)	63 (10)	13 (2)	88 (14)	4.2 [0.7;25.26]
Simple reaction time[d]	33 (6)	67 (12)	25 (2)	75 (6)	1.5 [0.23;9.8]	27 (4)	73 (11)	36 (4)	64 (7)	0.64 [0.12;3.41]
Selective attention[d]	25 (4)	75 (12)	17 (2)	83 (10)	1.67 [0.25;11.07]	8 (1)	92 (11)	31 (5)	69 (11)	0.2 [0.02;2]
Inhibition[d]	38 (6)	63 (10)	33 (4)	67 (8)	1.2 [0.25;5.77]	27 (3)	73 (8)	41 (7)	59 (10)	0.54 [0.1;2.77]
Verbal memory[d]	57 (13)	43 (10)	63 (12)	37 (7)	0.76 [0.22;2.63]	65 (13)	35 (7)	55 (12)	45 (10)	1.55 [0.45;5.37]
Figural memory[d]	33 (5)	67 (10)	38 (3)	63 (5)	0.83 [0.14;4.99]	22 (2)	78 (7)	43 (6)	57 (8)	0.38 [0.06;2.53]
Fluency[c]	50 (10)	50 (10)	25 (5)	75 (15)	3 [0.79;11.44]	30 (6)	70 (14)	45 (9)	55 (11)	0.52 [0.14;1.92]

[a] Cognitive dysfunction according to cut-off-values [28, 30] defined as standard value ≤ 93, standard value ≤ 88, standard value ≤ 85 respective percentile rank ≤ 20, percentile rank ≤ 16, percentile rank ≤ 10
[b] Cognitive dysfunction defined as standard value ≤ 93 respective percentile rank ≤ 20
[c] Cognitive dysfunction defined as standard value ≤ 88 respective percentile rank ≤ 16
[d] Cognitive dysfunction defined as standard value ≤ 85 respective percentile rank ≤ 10
