# Supplementary material for: Mid-term treatment-related cognitive sequelae in glioma patients
Source: J Neurooncol. 2022 Jul 7;159(1):65–79. doi: 10.1007/s11060-022-04044-1 (PMC9325813; doi:10.1007/s11060-022-04044-1)
Supplement: Supplementary file 4 — Supplementary file4 (PDF 441 KB) [file 11060_2022_4044_MOESM4_ESM.pdf]

Supplementary material (Online Resource 4)

Manuscript title:	Mid-term treatment-related cognitive sequelae in glioma patients
Journal:	Journal of Neuro-Oncology
Authors:	Sabine Schlömer, Jörg Felsberg, Milena Pertz, Bettina Hentschel, Markus Löffler, Gabriele Schackert, Dietmar Krex, Tareq Juratli, Joerg Christian Tonn, Oliver Schnell, Hartmut Vatter, Matthias Simon, Manfred Westphal, Tobias Martens, Michael Sabel, Martin Bendszus, Nils Dörner, Klaus Fliessbach, Christian Hoppe, Guido Reifenberger, Michael Weller, Uwe Schlegel
Corresponding author:	Sabine Schlömer
Affiliation of corresponding author:	Department of Neurology, University Hospital Knappschaftskrankenhaus, Ruhr University Bochum,<br>In der Schornau 23-25, D-44892 Bochum, Germany
E-Mail address of corresponding author:	sabine.schloemer@kk-bochum.de

Table ESM-4 Neurocognitive change within and between histopathological groups. T-test statistics for mean extent of cognitive improvement (i.e. mean difference [M_{diff}, in percentile ranks]) between baseline and follow-up neuropsychological assessment, separated for patients with oligodendroglioma and astrocytoma. Asterisks indicate statistically significant cognitive improvement. ANOVA (with F-statistics) for differences in cognitive change between histopathological groups. CI confidence interval, df degree of freedom. * $p < .05$, ** $p < .01$

	**Oligodendroglioma (n = 18)**				**Astrocytoma (n = 28)**				**ANOVA**		
	M_{diff} (95% CI)	t-value	df	p-value	M_{diff} (95% CI)	t-value	df	p-value	F-value	df	p-value
Short-term memory	10.7 (-3.1-24.5)	1.63	17	.121	9.8 (-0.3-19.8)	2.0	27	.056	0.01	1,45	.910
Working memory	20.4 (-1.4-42.3)	1.98	17	.065	2.7 (-13.4-18.8)	0.35	27	.732	1.91	1,45	.174
Simple reaction time	18.8 (1.9-35.6)	2.35	17	.031*	4.4 (-7.0-15.7)	0.79	27	.438	2.35	1,45	.133
Selective attention	22.8 (5.7-39.9)	2.81	17	.012*	17.7 (5.9-29.6)	3.07	26	.005**	0.27	1,44	.606
Inhibition	26.7 (12.6-40.7)	4.01	17	.001**	6.4 (-6.2-19.0)	1.05	26	.304	4.79	1,44	.034*
Verbal memory	14.7 (2.8-26.5)	2.60	17	.019*	13.9 (2.2-25.6)	2.46	25	.021*	0.01	1,43	.929
Figural memory	13.8 (-1.6-29.2)	1.92	14	.075	12.8 (0.4-25.2)	2.12	25	.044*	0.01	1,40	.915
Fluency	7.2 (-10.1-24.4)	0.88	17	.393	8.9 (-1.9-19.6)	1.69	27	.104	0.03	1,45	.856
