# Supplementary material for: Mid-term treatment-related cognitive sequelae in glioma patients
Source: J Neurooncol. 2022 Jul 7;159(1):65–79. doi: 10.1007/s11060-022-04044-1 (PMC9325813; doi:10.1007/s11060-022-04044-1)

Supplementary material (Online Resource 5)

Manuscript title:	Mid-term treatment-related cognitive sequelae in glioma patients
Journal:	Journal of Neuro-Oncology

Authors:	Sabine Schlömer, Jörg Felsberg, Milena Pertz, Bettina Hentschel, Markus Löffler, Gabriele Schackert, Dietmar Krex, Tareq Juratli, Joerg Christian Tonn, Oliver Schnell, Hartmut Vatter, Matthias Simon, Manfred Westphal, Tobias Martens, Michael Sabel, Martin Bendszus, Nils Dörner, Klaus Fliessbach, Christian Hoppe, Guido Reifenberger, Michael Weller, Uwe Schlegel
Corresponding author:	Sabine Schlömer
Affiliation of corresponding author:	Department of Neurology, University Hospital Knappschaftskrankenhaus, Ruhr University Bochum, In der Schornau 23-25, D-44892 Bochum, Germany
E-Mail address of corresponding author:	sabine.schloemer@kk-bochum.de

Fig. ESM-5 Cognitive performance (in percentile ranks) in NeuroCog FX subtests at baseline and follow-up, separated for histopathological groups (oligodendroglioma [a and c] vs. astrocytoma [b and d]) and dichotomized treatment groups (with radiotherapy [RT+] vs. without radiotherapy [RT-] and with chemotherapy [ChT+] vs. without chemotherapy [ChT-]). Asterisks indicate statistically significant improvement of cognitive functioning between baseline and follow-up. Bars indicate standard error of mean. * $p < .05$, ** $p < .01$.

a) Oligodendroglioma, n = 4 RT+ and n = 14 RT-

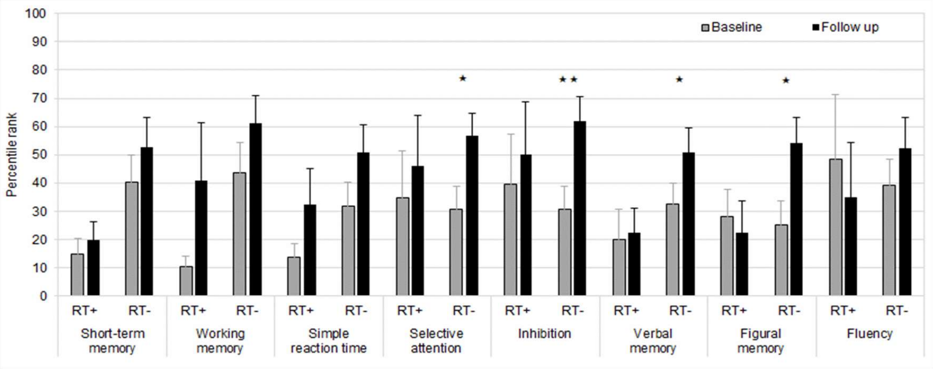

b) Astrocytoma, n = 11 RT+ and n = 17 RT-

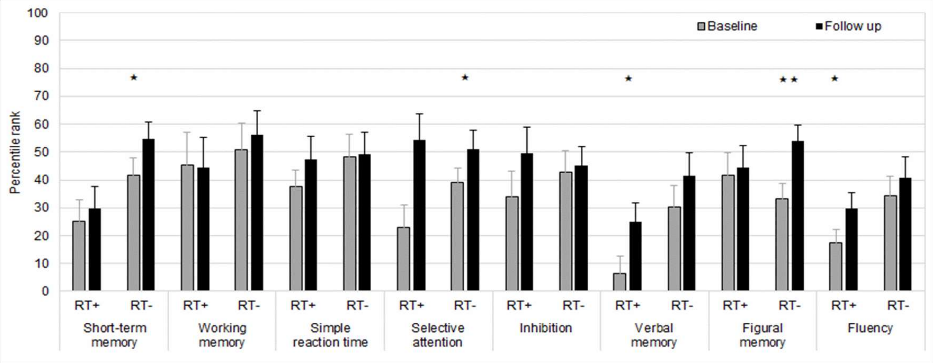

c) Oligodendroglioma, n = 13 ChT+ and n = 5 ChT-

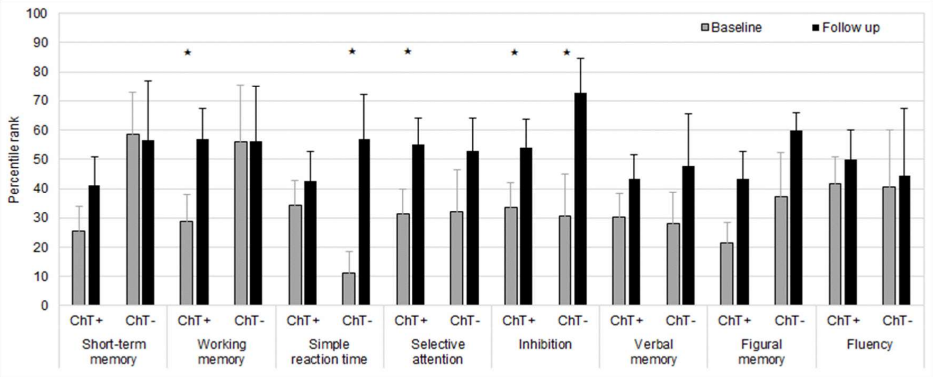

d) Astrocytoma, n = 14 ChT+ and n = 14 ChT-

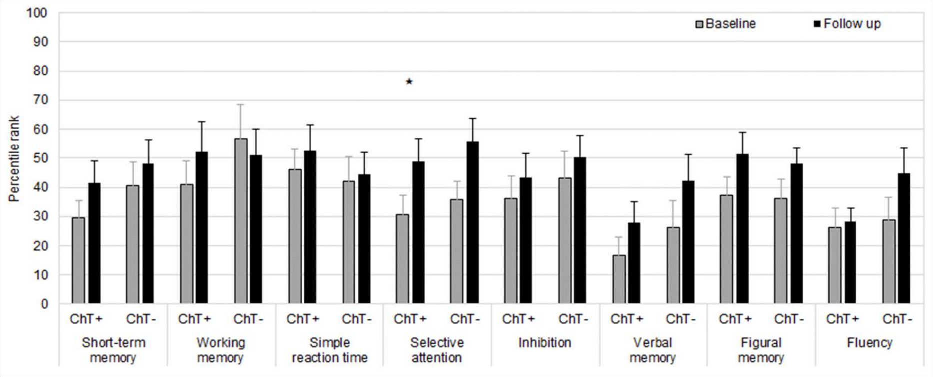

Supplement: Supplementary file 5 — Supplementary file5 (PDF 819 KB) [file 11060_2022_4044_MOESM5_ESM.pdf]
