# Supplementary material for: Mid-term treatment-related cognitive sequelae in glioma patients
Source: J Neurooncol. 2022 Jul 7;159(1):65–79. doi: 10.1007/s11060-022-04044-1 (PMC9325813; doi:10.1007/s11060-022-04044-1)
Supplement: Supplementary file 6 — Supplementary file6 (PDF 508 KB) [file 11060_2022_4044_MOESM6_ESM.pdf]

Supplementary material (Online Resource 6)

Manuscript title:	Mid-term treatment-related cognitive sequelae in glioma patients
Journal:	Journal of Neuro-Oncology
Authors:	Sabine Schlömer, Jörg Felsberg, Milena Pertz, Bettina Hentschel, Markus Löffler, Gabriele Schackert, Dietmar Krex, Tareq Juratli, Joerg Christian Tonn, Oliver Schnell, Hartmut Vatter, Matthias Simon, Manfred Westphal, Tobias Martens, Michael Sabel, Martin Bendszus, Nils Dörner, Klaus Fliessbach, Christian Hoppe, Guido Reifenberger, Michael Weller, Uwe Schlegel
Corresponding author:	Sabine Schlömer
Affiliation of corresponding author:	Department of Neurology, University Hospital Knappschaftskrankenhaus, Ruhr University Bochum, In der Schornau 23-25, D-44892 Bochum, Germany
E-Mail address of corresponding author:	sabine.schloemer@kk-bochum.de

Table ESM-6 Neurocognitive change within and between patients with tumor resection and patients with biopsy. T-test statistics for mean extent of cognitive improvement (i.e. mean difference [M_{diff}, in percentile ranks]) between baseline and follow-up neuropsychological assessment, separated for resected patients (patients with total, subtotal or partial resection) and patients with biopsy. Asterisks indicate statistically significant cognitive improvement. ANOVA (with F-statistics) for differences in cognitive change between groups. CI confidence interval, df degree of freedom. * $p < .05$, ** $p < .01$, *** $p < .001$

	**Patients with resection (n = 70)**				**Patients with biopsy (n = 16)**				**ANOVA**		
	M_{diff} (95% CI)	t-value	df	p-value	M_{diff} (95% CI)	t-value	df	p-value	F-value	df	p-value
Short-term memory	11.7 (5.0-18.3)	3.50	69	.001**	6.1 (-12.8-24.9)	0.69	15	.504	0.48	1,85	.493
Working memory	18.9 (7.6-30.1)	3.35	69	.001**	16.5 (-2.7-35.7)	1.84	14	.087	0.03	1,84	.853
Simple reaction time	14.3 (6.7-22.0)	3.76	68	<.001***	6.4 (-7.0-19.9)	1.02	15	.323	0.87	1,84	.355
Selective attention	22.0 (14.4-29.7)	5.77	67	<.001***	21.5 (4.2-38.8)	2.66	14	.019*	0.01	1,82	.949
Inhibition	17.7 (9.5-26.0)	4.28	68	<.001***	13.4 (-0.5-27.3)	2.07	14	.057	0.21	1,83	.646
Verbal memory	12.8 (6.5-19.0)	4.10	67	<.001***	5.9 (-6.2-18.0)	1.03	15	.318	0.97	1,83	.328
Figural memory	16.5 (9.4-23.6)	4.65	62	<.001***	13.6 (0.1-27.1)	2.16	14	.048*	0.13	1,77	.715
Fluency	14.8 (7.1-22.4)	3.84	69	<.001***	7.3 (-11.3-25.8)	0.85	13	.411	0.63	1,83	.429
